# Performance of woven fabrics for absorbent applications

**DOI:** 10.1038/s41598-026-41834-3

**Published:** 2026-03-23

**Authors:** Wael A. Hashima, Manar Y. Abd El-Aziz, Mohamed Hakam, Eman Mustafa

**Affiliations:** 1 Textile Engineering Department, Faculty of Engineering, Alexandria University, Alexandria, Egypt; 2 Clothing and Knitting Industrial Research Department, Textile Research and Technology Institute, National Research Centre, 33 EL Bohouth st. (Former EL Tahrir st.), P.O.12622, Dokki, Giza Egypt; 3 Textile Department, Faculty of Technology and Education, Suez University, Suez, Egypt

**Keywords:** Cotton woven fabrics, Weft yarn, Roving, Moisture management, Absorbent textiles, Engineering, Materials science

## Abstract

This study investigates the performance of woven fabrics developed using modified cotton fabric, where the weft yarns consist of roving and the warp yarns are conventional spun yarns, aiming to enhance absorbency and moisture transport properties in functional absorbent textile products. Two weave structures - plain and twill - were produced with varying weft densities of 5, 7, and 9 picks/cm, using roving yarns with a yarn count of Ne 1.1 in the weft direction. A comprehensive set of standard tests was conducted, including air permeability, thermal conductivity, abrasion resistance, pilling grades, tensile strength, dimensional stability, fabric friction, fabric roughness, and moisture management performance. Two-way ANOVA was used to examine the relationships between fabric structure, pick density, and the measured performance properties, while a radar chart was employed to evaluate and compare the samples by integrating their physical, mechanical, and tactile characteristics. T9 (Twill – 9 picks/cm) ranked as the top-performing sample, followed by P9 (Plain – 9 picks/cm) and T5 (Twill – 5 picks/cm). However, based on moisture-management indicators, specifically the One-Way Transport Index (905.2%) and Overall Moisture Management Capacity (0.98), T5 showed the highest absorption efficiency, T5 optimizes moisture functionality, illustrating a trade-off between mechanical/tactile properties and moisture-management performance.

## Introduction

The performance of absorbent fabrics is determined by a combination of structural, mechanical, and comfort-related properties that collectively influence their functionality in various end-use applications. Key characteristics - including thermal, mechanical, tactile properties, and moisture management performance - play essential roles in defining how these fabrics behave under conditions involving liquid exposure, skin contact, or mechanical stresses^1^. Each property contributes to specific aspects of comfort, durability, and hygienic performance, making the optimization of absorbent fabrics critical for applications ranging from personal care and medical products to household and technical textiles. These diverse requirements highlight the importance of enhancing the overall performance of absorbent fabrics to ensure they meet the functional, comfort, and hygiene needs across a wide spectrum of user scenarios^2,3^.

In addition, absorbent textiles are increasingly integrated into functional clothing, such as moisture-regulating thermal jackets, which rely on inner woven layers to wick sweat away from the body and maintain thermal comfort^4,5^. This moisture management function is also essential in sportswear, and medical dressings, where effective fluid handling plays a key role in enhancing comfort, performance, and hygiene^6,7^.

These diverse applications demonstrate the importance of optimizing the moisture management performance of woven pads to meet the functional, comfort, and hygiene needs across various user scenarios. Continued innovation in fabric construction, fiber selection, and finishing techniques is therefore essential for the advancement of these multifunctional absorbent textile solutions.

Cotton remains one of the most widely used natural fibers in moisture management textiles due to its inherent comfort, breathability, and skin compatibility. Its hydrophilic nature allows it to absorb large amounts of moisture, making it a suitable candidate for absorbent products such as reusable sanitary pads, face towels, and undergarments^8^. However, conventional cotton fabrics have limitations in moisture transport and drying speed, often leading to a wet and clingy feel during extended wear^9^. To address these issues, researchers have investigated various strategies to enhance cotton’s moisture handling performance. These include modifying yarn structures, incorporating functional finishes, and blending cotton with synthetic or high-performance fibers to improve wicking and drying behaviour. Despite the emergence of synthetic alternatives, cotton remains highly valued for its biodegradability, renewability, and non-irritating properties, especially in products designed for direct skin contact. As a result, optimizing cotton for efficient moisture management remains a key research direction in the development of sustainable and functional absorbent textile products^10,11^.

Considering cotton’s natural advantages - such as softness, breathability, and biocompatibility - this study aims to retain these desirable properties while significantly enhancing the fabric’s moisture management ability and absorbency performance. The current research focuses on the development of a modified cotton woven fabric^12–14^, in which the weft yarn (picks) is replaced with roving yarn instead of conventional spun yarn. Roving, being less twisted and bulkier than standard yarns, offers a higher surface area and increased porosity, which can promote faster moisture uptake and more efficient distribution across the fabric structure. By incorporating roving yarns into the weft direction, the fabric is expected to achieve improved fluid absorption dynamics without compromising the inherent comfort and sustainability of cotton. This approach reflects an innovative strategy for enhancing the functional performance of cotton-based absorbent textiles.

## Materials and methods

### Materials used

Fabric samples were produced using rapier looms, with roving employed as the weft yarn in the fabric. Both the warp yarns and the roving were composed of 100% Egyptian G86 cotton fibres. Roving count was 1.1 Ne with 0.8 turns/cm. The roving was rewound onto a cone package to ensure compatibility with the creel system of the weaving machine. Table 1 presents the specifications of six different fabric samples, all featuring the same weft but varying in fabric structure and pick density. The samples included two types of fabric structures - plain (1/1) and twill (2/2) - with three different pick densities: 5, 7 and 9 picks/cm. The warp yarns remained consistent across all fabric samples, with a warp count of 20/2 Ne and a warp density of 9 ends/cm.

**Table 1:** Fabric specification.

Sample code	Structure	Picks/cm	Weft count (Ne)	Turns/cm	Thickness (mm)	g/m^2^
P5	Plain	5	1.1	0.8	1.07	387
P7	Plain	7	1.1	0.8	1.11	458
P9	Plain	9	1.1	0.8	1.12	545
T5	Twill	5	1.1	0.8	1.28	388
T7	Twill	7	1.1	0.8	1.33	459
T9	Twill	9	1.1	0.8	1.66	625

### Testing methods

Textile samples were washed according to ISO 6330 standard, using Type A washing conditions. The procedure was followed by flat drying to maintain dimensional stability. Reference Detergent 3 was used during the washing process to ensure standardized cleaning performance. This method ensures repeatable and comparable results in evaluating fabric behaviour after laundering. All test specimens were preconditioned at ambient conditions (20 °C ± 2 and 65% ± 2 RH%) according to ISO 139:2005. This conditioning procedure was applied to all tests conducted.

Fabric thickness was measured by using the SDL fabric digital thickness tester thickness tester in accordance with the ISO 5084 standard test.

Air permeability was evaluated following the ASTM D737 standard test using an SDL ATLAS M021 tester. Thermal conductivity was determined with the KES-F7 Thermo Labo apparatus, ensuring a constant temperature difference between the two plates during measurement^15^.

Pilling grades were evaluated following the ASTM D4970 standard method after subjecting the fabrics to 2000 cycles using standard wool abradant fabric^16^. The weight loss percentage, reflecting abrasion resistance, was measured after 5000 cycles using a Martindale Abrasion tester (M235)^17^. The flexural rigidity of the fabric samples was evaluated according to ISO 9073-7, which provides a standardized method for determining bending length using the cantilever principle. The tensile strength and elongation percentage of the fabric specimens were determined using an Instron tester (USA, 2005) in accordance with the ASTM D1683/D1683M-22 standard test method.

Dimensional stability of the specimens was evaluated in accordance with ISO 5077:2007, which specifies the method for determining dimensional changes in fabrics after domestic washing and drying. Test specimens were preconditioned under standard atmospheric conditions, washed and dried when considering ISO 139:2005 and ISO 6330.

The surface mean deviation (SMD, µm) and mean frictional coefficient (MIU) were measured using the KES-F module (Kawabata, 2020) to quantify fabric roughness and fabric friction, respectively^18^.

**Fig. 1:**
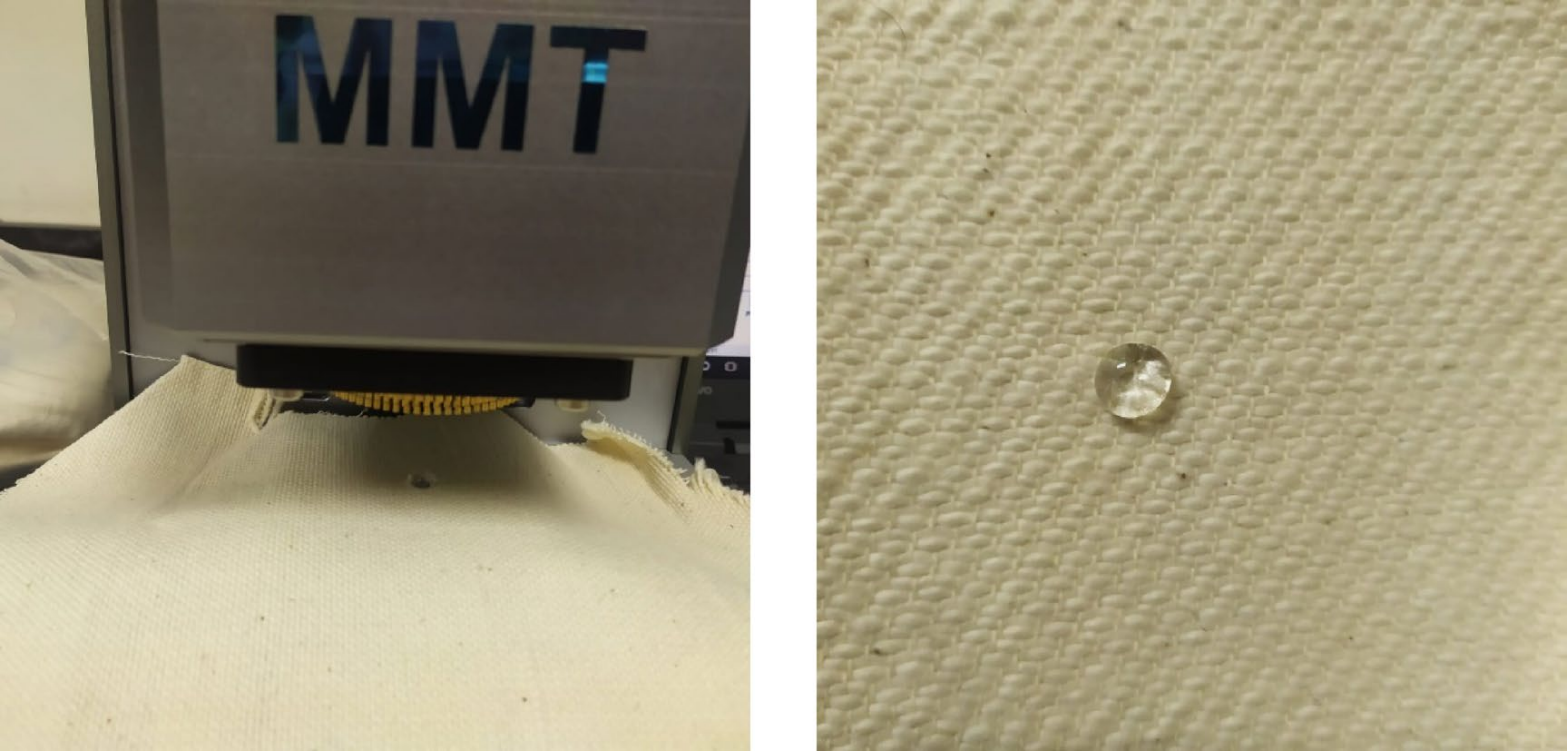
Fabric sample setup of moisture management tester.

The liquid moisture management properties of fabric samples were measured according to.

to the AATCC 195 Liquid Moisture Management Test [AATCC, 2011]. Specimens (8 × 8 cm) were prepared after laundering according to AATCC LP1 and conditioned at 21 ± 2 °C and 65 ± 5% relative humidity. Testing was performed using a 9% sodium chloride (NaCl) solution in distilled water. This method involves placing the fabric specimens between sensors to evaluate their liquid moisture management performance based on changes in electrical resistance, as shown in (Fig. 1). The upper sensor contacts the top surface of the sample, which represents the side facing the wearer’s skin. The bottom surface of the fabric sample, which corresponds to the outer fabric layer, contacts the lower sensor^19^.

### Data analysis

Data obtained from different testing methods were analysed both statistically and graphically. Bar charts were used to illustrate the effect of various variables on the fabric’s physical and mechanical properties.

A two-way ANOVA with replications was performed to evaluate the simultaneous effects of fabric structure and Picks/cm on the measured properties, while accounting for variability between replicates. Five samples were tested for each property, which ensures statistical reliability of the ANOVA results. This statistical approach allowed for the assessment of main effects and interaction effects between the two factors, providing a robust understanding of their influence on the observed fabric properties.

A radar chart is used to compare the physical, mechanical and tactile properties across the fabric samples simultaneously, allowing for a clear visualization of overall performance patterns. Regarding the fabric moisture performance, the Overall Moisture Management Capacity (OMMC) is considered the most comprehensive indicator which integrates absorption rate, spreading speed, and one-way transport capability into a single evaluative parameter. Higher OMMC values indicate superior overall moisture management behaviour.

## Results and discussion

### Fabric air permeability and thermal conductivity

**Fig. 2:**
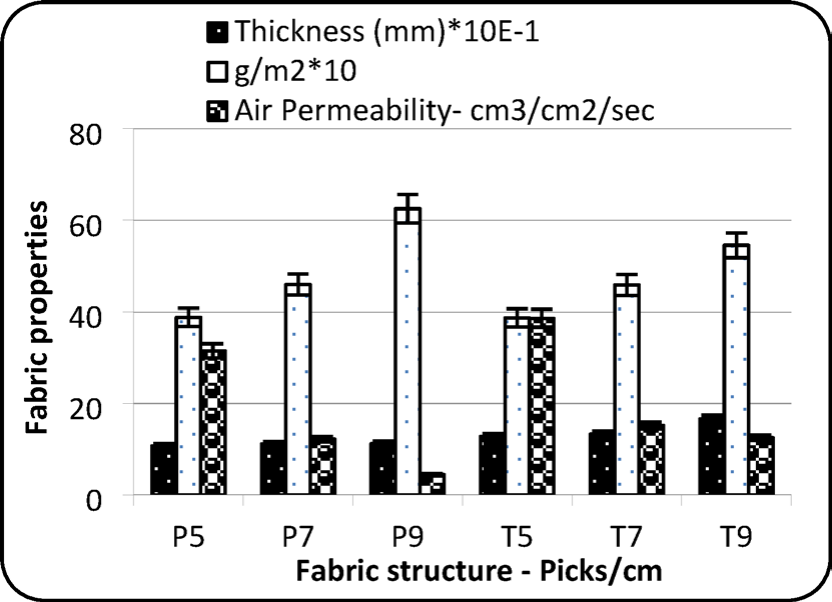
Air permeability.

The results indicate that air permeability is significantly affected by fabric structure and pick density, as shown by P-values below 0.05 in Table 1. Figure 2 shows that fabrics with a twill weave structure exhibited higher air permeability compared to plain weave structures. This can be attributed to the less compact nature of twill weaves, which contain longer floats and fewer interlacing, allowing more air to pass through the fabric compared to tightly constructed plain weaves, especially in the warp direction^15^. Moreover, the plain weave forces the roving to flatten at every intersection (crimp exchange). Therefore, in P9 (high density), the roving likely creates a “seal”.

Additionally, Fig. 2 presents measurements of fabric thickness, weight (gsm), and air permeability of plain and twill samples. Across both weave types, there is a clear trend: as thickness and fabric weight increase, air permeability decreases. This inverse relationship suggests that higher fabric thickness and weight contribute to lower air permeability, likely due to a denser fabric structure that restricts airflow.

**Fig. 3:**
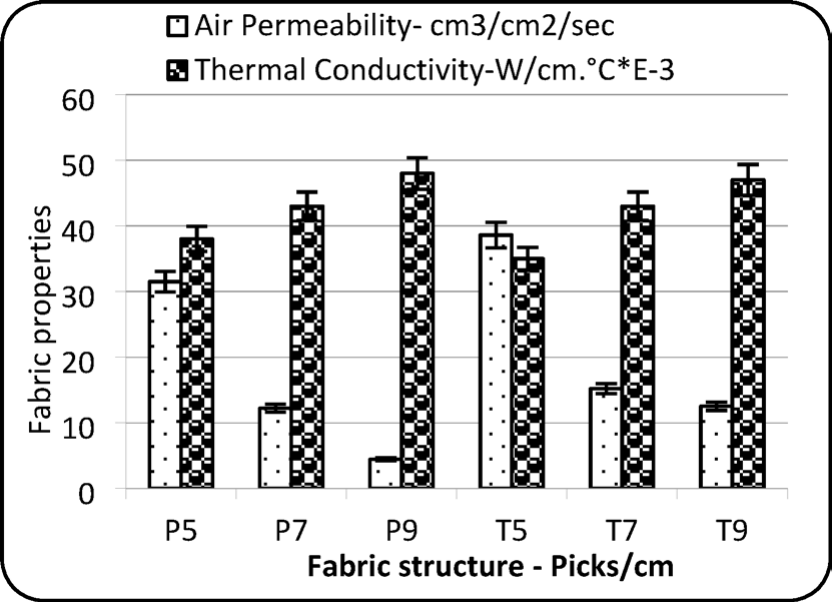
Thermal conductivity.

Table 2 demonstrates that the relationship between fabric structure and thermal conductivity is statistically significant according to *P*-values < 0.05. As shown in Fig. 3, an inverse relationship was observed between thermal conductivity and air permeability. Fabrics with higher thermal conductivity exhibited reduced air permeability, which can be attributed to increased fiber contact and lower porosity. Higher fabric density (picks/cm) leads to structural tightening and reduced inter-yarn porosity. consequently, the increased yarn interlacements and contact areas promote conductive heat transfer through the fabric structure, thereby increasing thermal conductivity^20^.

### Pilling grades and abrasion weight loss%

**Fig. 4:**
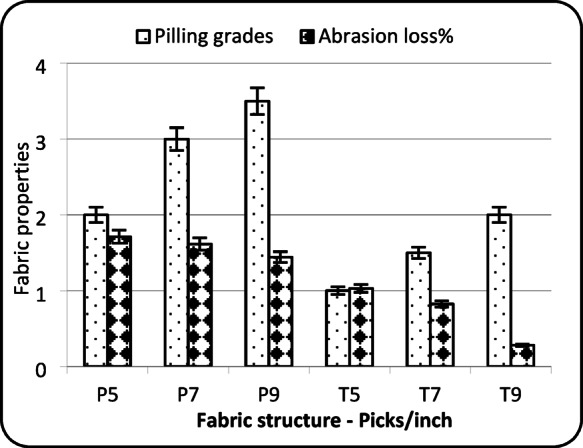
Pilling grades and abrasion loss.

The data presented in Table 2 show that both fabric structure and pick density have statistically significant effects on the pilling behaviour of the fabric. Figure 4 shows that plain weave fabrics consistently exhibit higher pilling grades compared to twill fabrics at the same pick densities. This suggests that plain weave offers better resistance to pilling. This can be attributed to the tighter and more interlaced structure of the plain weave, which limits fiber movement on the fabric surface, thereby reducing the formation of pills. Moreover, the increase in weft density makes the fabric more compact, which further enhances its resistance to pilling by minimizing surface fuzz and friction^16^.

Additionally, Fig. 4 shows that plain weave fabrics exhibit greater weight loss during abrasion tests. This aligns with the physical characteristics of the plain weave, which is generally tighter and rougher due to more frequent interlacing between warp and weft yarns. These frequent contact points create higher surface friction, making the fabric more prone to wear under abrasion^21^.

However, as pick density increases from 5 to 9 picks/cm, abrasion weight loss% decreases in both weaves, indicating that a higher weft density contributes to a more compact structure, enhancing the fabric’s resistance to abrasion^21^.

In contrast, pilling grades show an inverse pattern relative to abrasion loss. Fabrics with higher abrasion loss (such as plain weave) also exhibit higher pilling grades, suggesting better pilling resistance. This can be explained by the fact that cotton plain weaves are more susceptible to fiber breakage due to higher friction, which causes surface pills to wear off more quickly, thereby improving the pilling grades^16,17^.

### Flexural rigidity with warp and weft directions

**Fig. 5:**
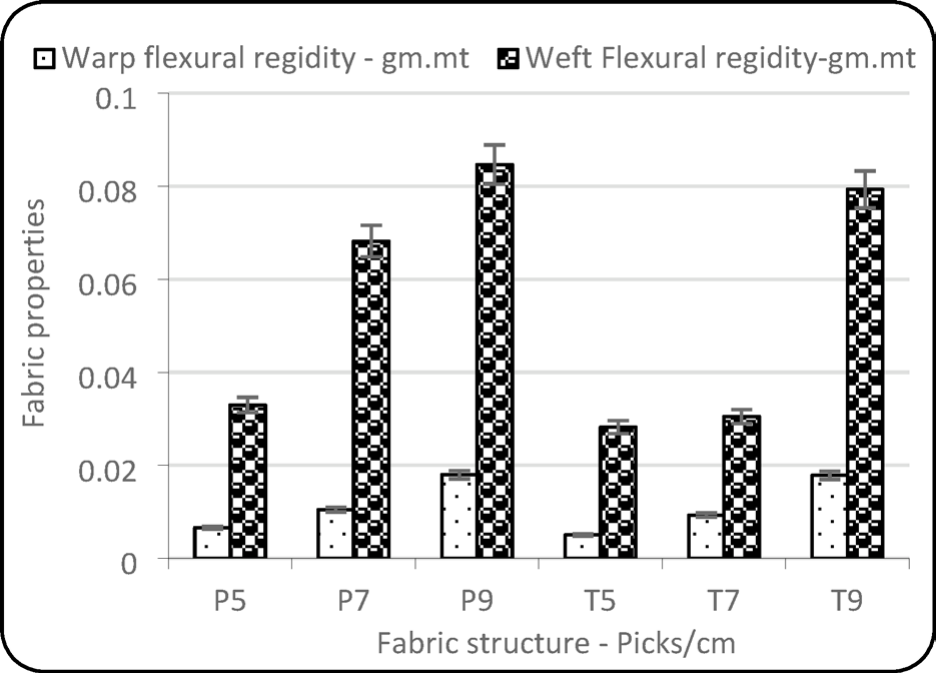
Flexural rigidity.

According to Table 2, pick density and fabric structure show a significant impact on warp rigidity and weft flexural rigidity. Figure 5 illustrates that flexural rigidity in the warp direction shows a consistent increase with the rise in pick density across both plain and twill weave structures. Moreover, with plain weave fabrics consistently exhibiting higher warp rigidity than twill fabrics at comparable pick densities, due to the tighter interlacement and more frequent yarn crossings in plain weave^22^. In the weft direction, the flexural rigidity also increases with pick density. The plain weave again demonstrated higher weft rigidity than the twill, highlighting the influence of structural tightness^23^.

### Fabric tensile strength in warp and weft directions

**Fig. 6:**
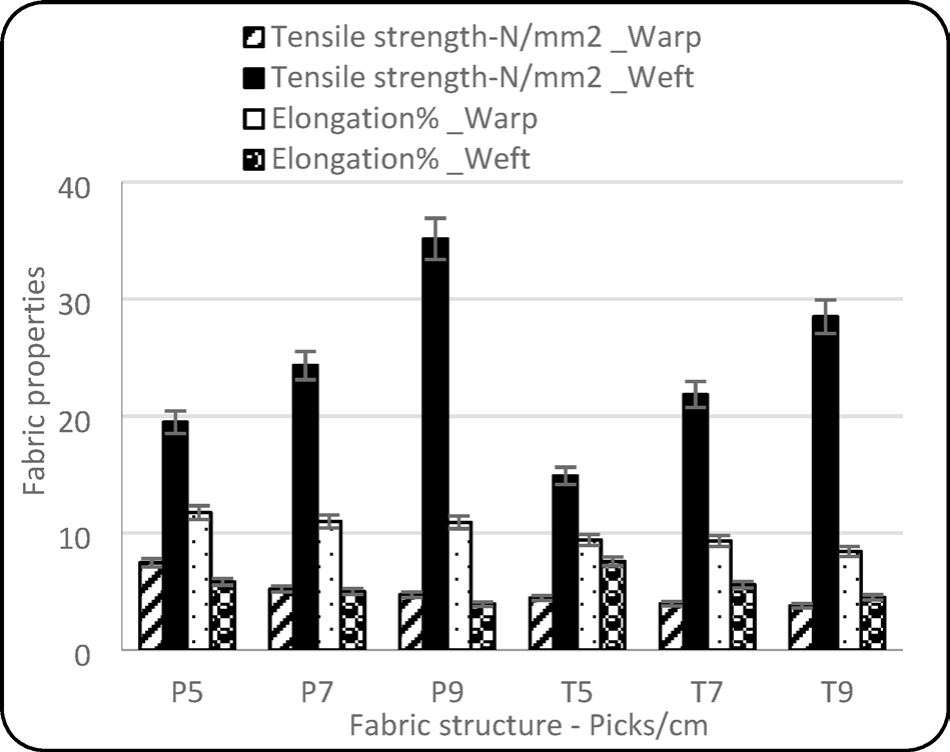
Tensile strength and elongation%.

Table 1 demonstrates that both pick density and fabric structure significantly influence the tensile strength and elongation in both the warp and weft directions. Moreover, Fig. 6 illustrates that the warp tensile strength decreases with increasing pick density in both plain and twill weaves. This decline is attributed to the higher crimp and geometric distortion in high-density fabrics create stress concentrations. Moreover, plain weave consistently exhibits higher tensile strength compared to twill, which is likely due to its higher number of interlacements that improve yarn binding and structural integrity^24^.

Figure 6 illustrates that higher pick density and plain weave structure exhibited higher tensile strength in the weft direction. As pick density increases, the number of weft yarns per unit length rises, resulting in greater material compactness and improved distribution of tensile loads during testing. This leads to enhanced resistance against weft-direction elongation and failure. Additionally, plain weave structures consistently exhibit higher tensile strength than twill at equivalent pick densities, which is attributed to their higher degree of interlacement. The frequent intersections between warp and weft in plain weaves increase the frictional binding and mechanical stability of the fabric, allowing it to better resist deformation^25^.

The statistical results presented in Table 1 indicate a significant relationship between fabric parameters and elongation in both the warp and weft directions. Figure 6 shows that plain weave exhibits higher warp elongation than twill weaves. This result can be attributed to the higher degree of yarn crimp typically found in plain structures. In plain weave, each warp yarn interlaces with every weft yarn in a one-over-one-under pattern, resulting in more frequent interlacements and higher crimp of the yarns. This crimp must be straightened during tensile loading, contributing to greater elongation^26^. Furthermore, the consistent decrease in warp elongation with increasing pick density can be explained by the increased fabric compactness and yarn interlocking. As pick density increases, the spaces between weft yarns are reduced, and the yarns are more tightly packed. This tighter construction limits the mobility of the yarns under tensile stress, restricting their ability to deform and thus reducing elongation^27^.

Additionally, as the pick density increases, the fabric’s ability to elongate in the weft direction decreases. Moreover, twill weave exhibited higher elongation compared to plain weave. This may seem unexpected at first, as plain weaves are generally more tightly interlaced, which could imply more crimp and thus more extensibility. However, in the weft direction, the behaviour is affected not only by crimp but also by the mobility and straightness of weft yarns. In twill weaves, the lower frequency of interlacements allows the weft yarns to follow a more relaxed path with less inter-yarn friction and fewer binding points, which permits greater deformation under tensile loading. Conversely, plain weaves, with their one-over-one-under pattern, create a highly interlocked structure, which restricts the movement of weft yarns more severely. The result is a stiffer fabric in the weft direction, and thus lower elongation^28^.

### Dimension stability

**Fig. 7:**
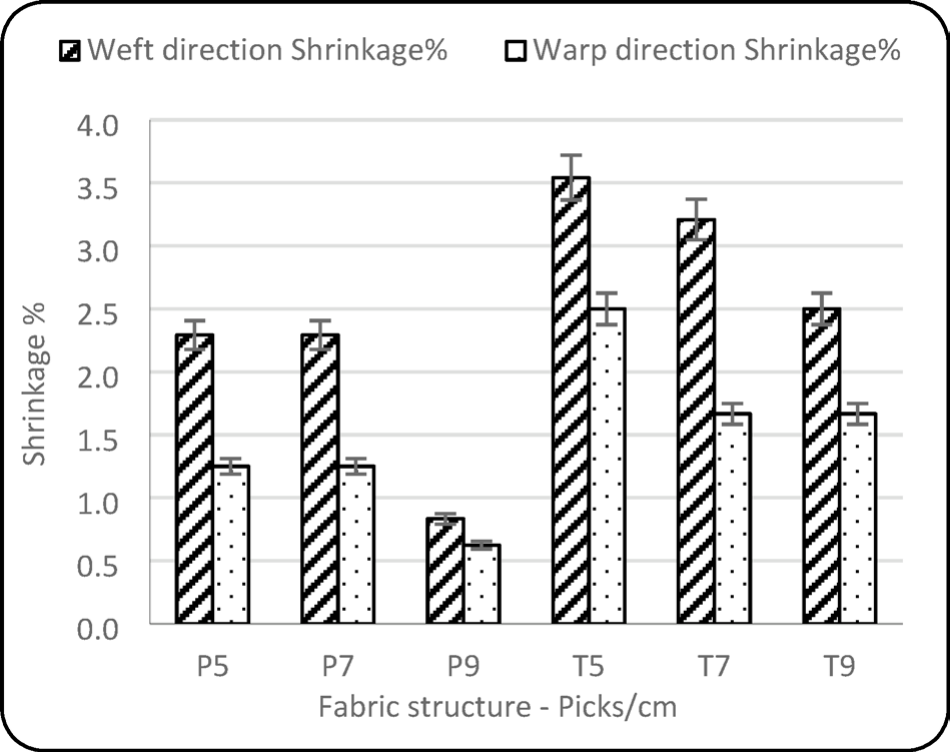
Dimension stability of fabric.

Table 2 shows that the statistical analysis of the weft direction shrinkage results highlights a statistically significant relationship between shrinkage and the fabric variables. Figure 7 demonstrates across all levels of pick density; plain weave fabrics consistently exhibit lower weft shrinkage compared to twill weaves. Additionally, as pick density increases, weft shrinkage decreases in both fabric types. The plain weave exhibited lower shrinkage in both directions due to its higher compactness and reduced yarn mobility compared to the twill weave^29^. Additionally, fabric variables significantly influence fabric shrinkage in the warp direction, with a similar trend observed for the lower shrinkage in the weft direction.

### Tactile properties

**Fig. 8:**
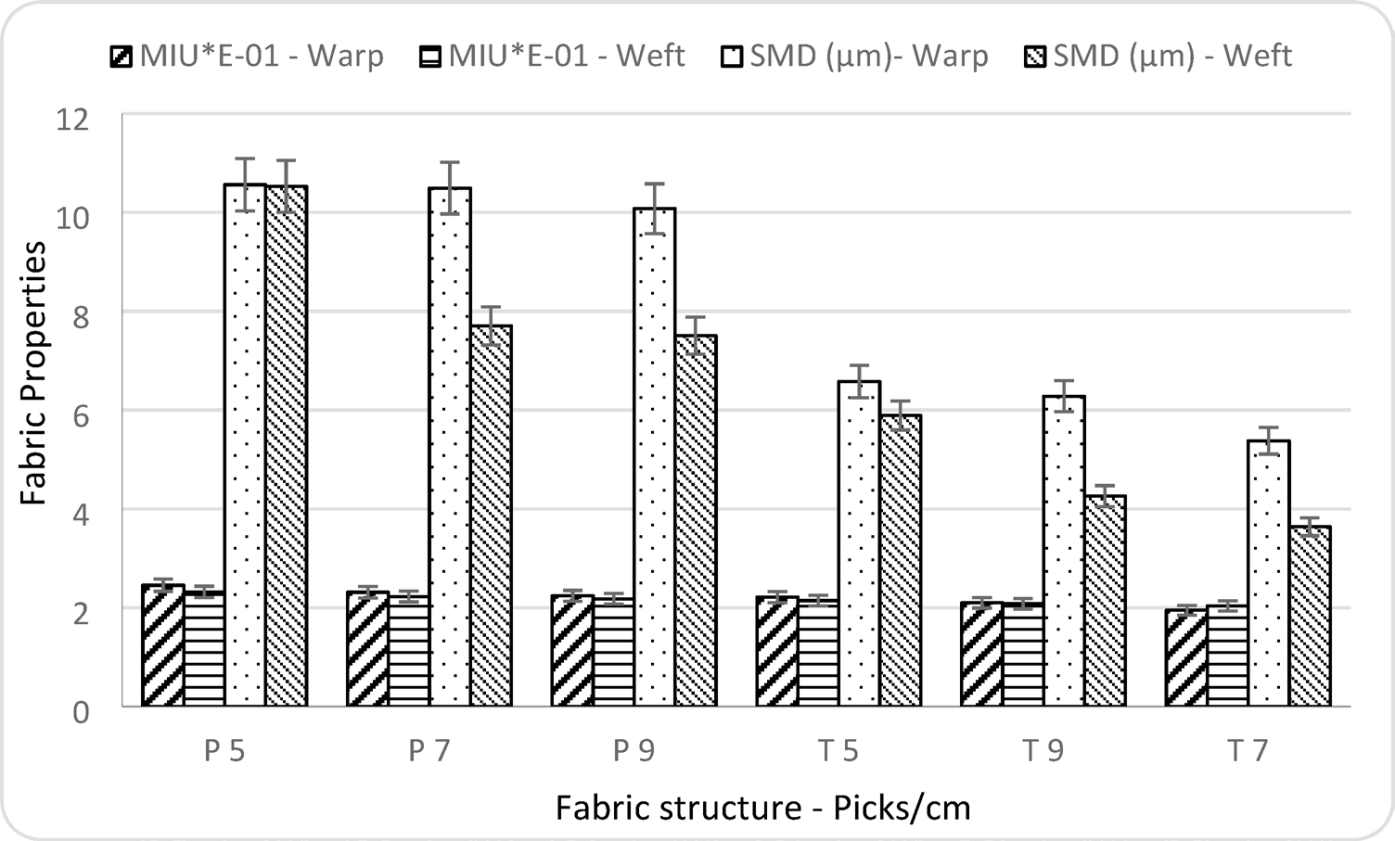
Fabric tactile properties with warp and weft directions.

Table 2 and Fig. 8 examine the effect of fabric structure and pick density on the tactile properties of the fabrics, represented by surface roughness (SMD, µm) and friction coefficient (MIU). The results demonstrate that both fabric structure and pick density significantly influence the tactile properties, with* p*-values below 0.05, confirming statistical significance at the 5% level. Higher amplitudes of (SMD - µm) of plain weave pattern were demonstrated due to more interlacing between yarns with the plain weave and more crowns which make the fabric surface rough compared to twill pattern. Because of the fabric surface of plain weave is rough, fabric friction (MIU) increased^30^. Higher pick density of plain and twill patterns registered lower values of (SMD - µm) and (MIU) because of more picks/cm decrease spaces between yarns which leads to a decrease in vertical deviations of the fabric surface.

**Table 2:** *P*-values for physical, mechanical and tactile properties.

Fabric characteristics	Fabric structure	Picks/cm	Interaction
Air Permeability cm^3^/cm^2^/sec	0.011	0.006	0.03
Thermal Conductivity W/cm °C	0.0001	0.000	0.002
Pilling grades	0.002	0.000	0.01
Abrasion loss%	0.005	0.000	0.008
Warp flexural rigidity—gm.mt	0.009	0.000	0.02
Weft flexural rigidity—gm.mt	0.02	0.03	0.04
Warp tensile strength (N/mm^2^)	0.002	0.041	0.043
Weft tensile strength (N/mm^2^)	0.000	0.000	0.005
Warp Elongation%	0.01	0.013	0.02
Weft Elongation%	0.000	0.000	0.006
Weft Shrinkage%	0.002	0.000	0.007
Warp Shrinkage%	0.011	0.03	0.04
MIU - Warp	0.012	0.000	0.02
MIU - Weft	0.000	0.021	0.033
SMD (µm)- Warp	0.02	0.000	0.03
SMD (µm) - Weft	0.007	0.004	0.005

### Rank of samples according to Radar chart area

**Fig. 9:**
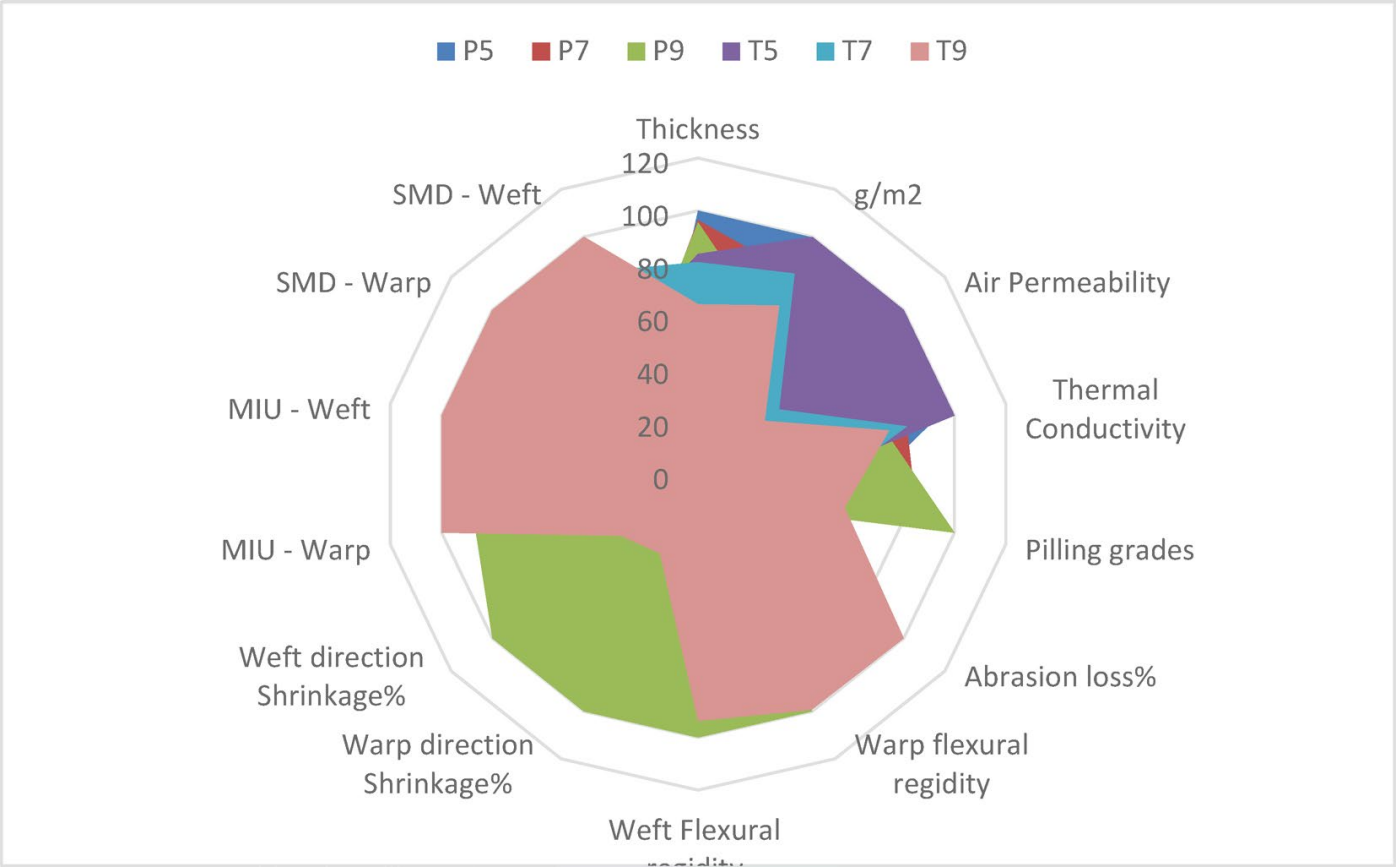
Radar chart of physical, mechanical and tactile properties.

As shown in Fig. 9, The radar chart presented a comprehensive comparative tool to evaluate the overall performance of the six fabric samples (P5, P7, P9, T5, T7, and T9) by integrating their physical, mechanical, and tactile properties into a single visual framework. In this representation, each axis corresponds to a specific performance parameter, and the enclosed radar area reflects the cumulative performance of the sample across all measured attributes. Accordingly, a larger radar area indicates a more balanced and superior overall performance profile. As shown in Table 3, T9 exhibited the largest radar area, ranking first, followed by P9, while P7 recorded the smallest area, ranking last. The higher ranking of T9 and P9 suggests that increasing pick density (9 picks per inch), particularly in the twill structure, contributed to enhanced overall functional performance. While T9 has the best aggregate physical/mechanical score, it may not be the optimal choice for the specific end-use of absorbency.

**Table 3:** Rank of samples according to Radar area.

Sample code	Radar area	Rank
P5	12521.8	Fifth
P7	12119.8	Sixth
P9	16848.7	Second
T5	13793.1	Third
T7	12786.7	Fourth
T9	18021.6	First

### Moisture management properties

The Moisture Management Test (MMT) evaluates a fabric’s ability to manage liquid absorption and movement, simulating situations where the material is exposed to fluids. Key parameters obtained include wetting time, which measures how quickly the fabric surface begins to absorb moisture, and absorption rate, indicating the speed at which liquid is drawn into the material. The maximum wetted radius reflects how far the moisture spreads from the point of contact, while spreading speed tracks the rate of that dispersion across both the top and bottom fabric surfaces. In addition, the one-way transport index quantifies the efficiency of moisture transfer from the inner to the outer surface, which is crucial for comfort. Lastly, the Overall Moisture Management Capacity (OMMC) provides a combined evaluation of all these factors, giving a comprehensive score of the fabric’s performance in moisture handling^31^.

Table 4 presents the statistical significance (p-values) of fabric structure, picks/cm, and their interaction on the measured moisture management properties. In general, the results indicate that both structural parameters exert a statistically significant influence on most moisture-related characteristics, as most p-values are below the significance level (*p* ≤ 0.05). This confirms that variations in weave structure and pick density, as well as their combined effect, play a decisive role in controlling liquid transport behaviour. However, certain parameters, such as maximum wetted radius (top and bottom surfaces), show no statistically significant differences, reflecting minimal influence of the examined variables.

**Fig. 10:**
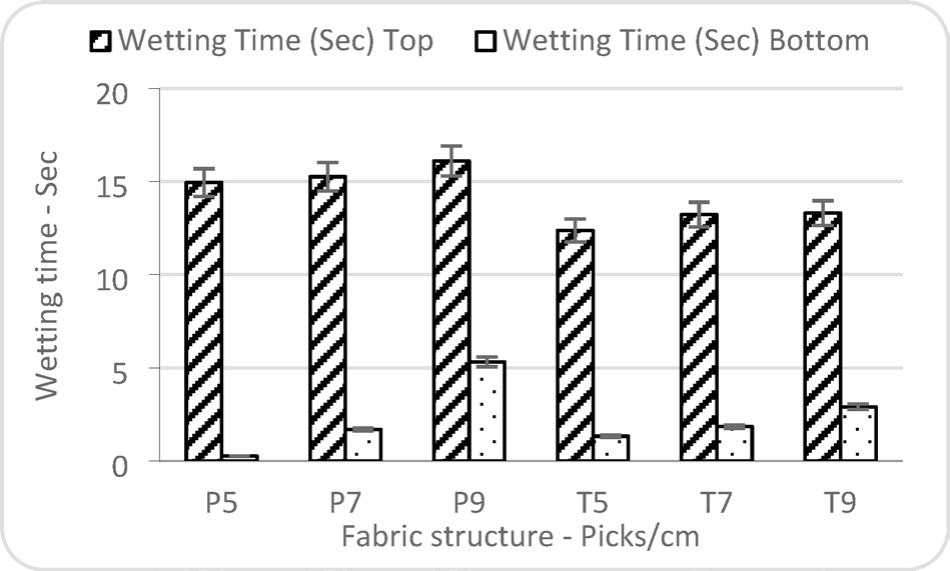
Factors affecting wetting time.

Figure 10 shows that increased weft density (picks/cm) contributes to longer wetting times of the top surface, possibly due to reduced pore space for liquid movement. The tighter interlacement in plain weaves likely hinders the initial penetration of moisture, resulting in delayed wetting. While both surfaces exhibit some degree of similarity in their moisture response, they are governed by different underlying mechanisms. The top surface, being directly exposed to the liquid source, primarily reflects the influence of surface morphology and porosity on initial absorption. In contrast, the wetting behaviour of the bottom surface is more affected by the fabric’s internal capillary network and its capacity to facilitate vertical moisture transport.

**Fig. 11:**
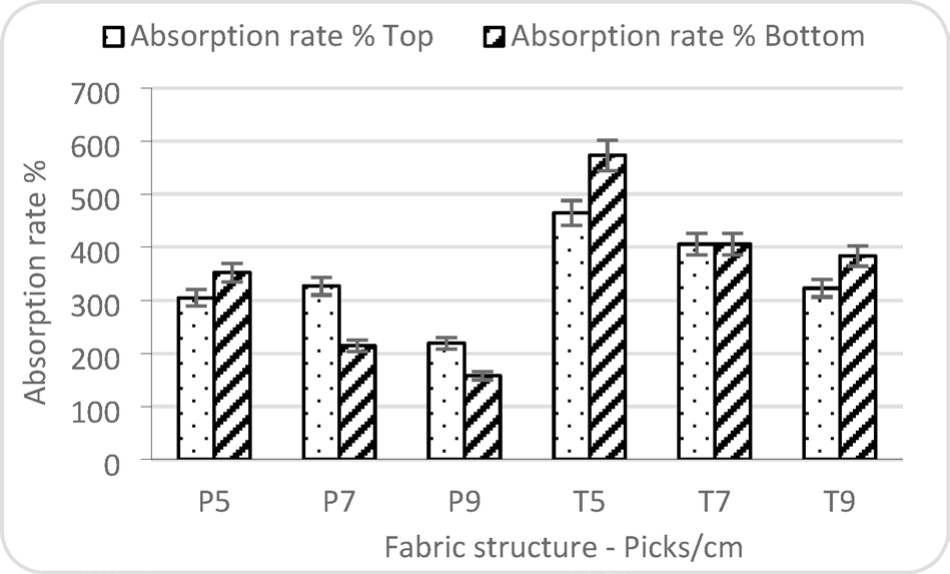
Factors affecting absorption rate.

Figure 11 illustrates that the absorption rate on both the top and bottom surfaces decreases with increasing weft density and when a plain weave structure is employed instead of a twill weave. The inverse relationship can be attributed to the structural characteristics of the fabrics. Plain weave, having a tighter interlacement and more compact structure, tends to limit the immediate uptake of moisture compared to the more open structure of twill fabrics. Similarly, increasing the number of picks per centimetre reduces the available pore space and capillary channels necessary for rapid moisture absorption. These results support the notion that more open weaves and lower weft densities promote faster moisture uptake at the fabric’s surface, which is critical for ensuring initial comfort in contact with liquid^32^. Consequently, twill weaves, with their more open structure, facilitate higher absorption rates on the bottom surface, promoting better moisture transport away from the skin. These results highlight the critical role of fabric architecture and yarn packing in governing moisture movement through the fabric thickness^33,34^.

**Fig. 12:**
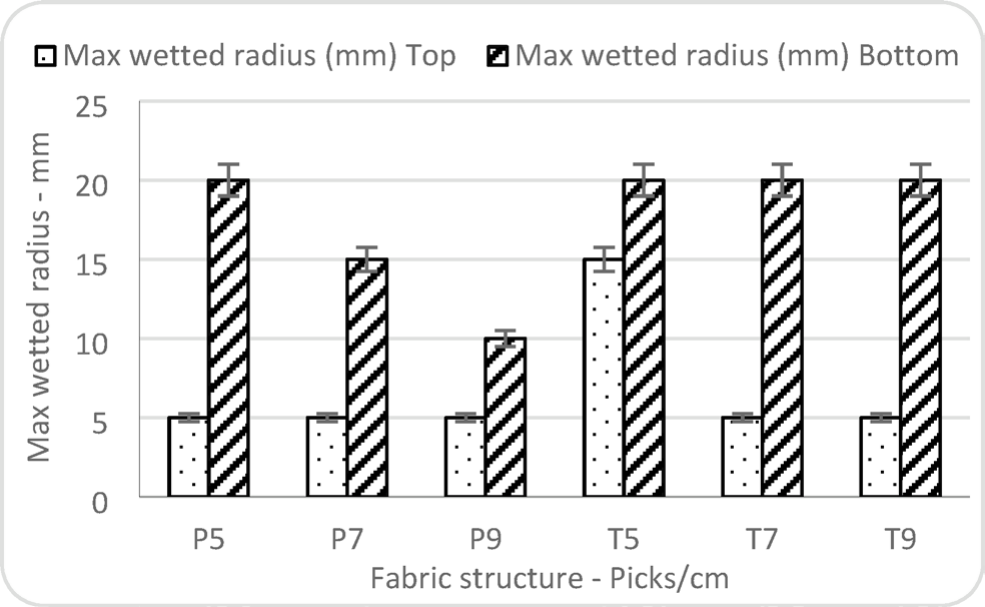
Factors affecting wetted radius.

Figure 12 showed that the maximum wetted radius on the top surface remained constant at 5 mm for five of the six fabric samples, irrespective of fabric structure (plain or twill) and weft density (picks/cm). However, a single deviation was observed in the twill fabric with a low weft density (5 picks/cm), which exhibited a wetted radius of 15 mm. This elevated value may be explained by the combined effect of the twill weave and the loose fabric construction^35^. Additionally, it is supported by its significantly higher absorption rate of the top surface (464%), as shown in Fig. (11), confirming that the increased wetted radius reflects a real moisture transport mechanism associated with the twill weave and low weft density. This structure promotes lateral liquid spreading prior to full thickness penetration, unlike the other samples which exhibit limited lateral wetting behaviour.

Twill fabrics, as shown in Fig. 12, exhibited a high wetted radius (20 mm) across all weft densities, indicating that bottom surface moisture transport remained unaffected by pick density in twill samples. In contrast, plain fabrics demonstrated a decrease in wetted radius with increasing picks/cm, from 20 mm at 5 picks/cm to 10 mm at 9 picks/cm. This trend suggests that the plain weave is more sensitive to changes in weft density, where higher density results in reduced moisture penetration or transport to the bottom surface.

Although the statistical significance was not achieved (*P*-values > 0.05), the trends observed indicate that plain fabrics, particularly with higher pick densities, limit the spread of moisture on the bottom surface. This may be due to their tighter structure at higher densities, which restricts vertical liquid transport. On the other hand, twill fabrics maintained a high wetted radius regardless of pick density, likely due to their floated yarns and more open interlacements, which facilitate greater moisture movement through the fabric’s thickness.

**Fig. 13:**
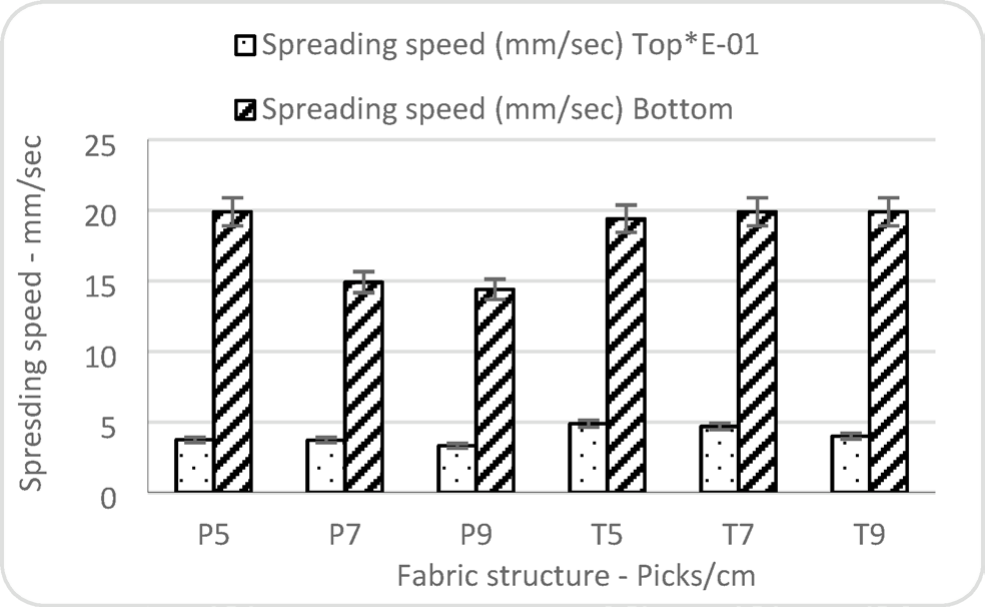
Factors affecting spreading speed.

Figure 13 shows that twill weaves, due to their float structure and more open interlacements, allow faster lateral movement of moisture according to the values of spreading speed. Higher weft density, particularly in plain weaves, results in a tighter structure, which impedes the flow of moisture on the surface. Twill fabrics and lower pick densities enhance moisture spreading, which is beneficial for rapid drying and comfort^36^. Moreover, plain fabrics exhibit a decrease in spreading speed with increasing picks/cm. This trend may be attributed to the increasing density in plain weaves, which restricts moisture mobility due to a tighter structure. In contrast, twill fabrics maintain consistently high spreading speeds across all densities, indicating that their structural openness and float yarns support uniform moisture transport through the fabric^37^.

**Table 4:** P-values for moisture management properties.

Fabric characteristics	Fabric structure	Picks/cm	Interaction
Wetting time (Sec)—Top	0.000	0.004	0.006
Wetting time (Sec)—Bottom	0.008	0.000	0.01
Absorption rate %—Top	0.000	0.032	0.035
Absorption rate %—Bottom	0.002	0.017	0.02
Max wetted radius—Top (mm)	0.42	0.5	0.57
Max wetted radius—Bottom (mm)	0.23	0.5	0.509
Spreading speed (mm/sec)—Top	0.022	0.03	0.04
Spreading speed (mm/sec)—Bottom	0.000	0.015	0.02
One way transport index %	0.001	0.002	0.007
OMMC	0.000	0.001	0.01

Table 5 presents the mean ± standard deviation values of OWTI and OMMC for each fabric, illustrating the effect of fabric structure (plain and twill weaves) and weft densities (5, 7, and 9 picks/cm) on moisture transport performance. The OWTI is a critical indicator of a fabric’s ability to transfer moisture from the inner (skin-facing) surface to the outer surface.

In the case of the plain weave, all OWTI values are negative, ranging from − 550.35% at a pick density of 5 to -317.22% at a pick density of 9. This clearly indicates that plain weave fabrics demonstrate poor one-way moisture transport, as negative values suggest moisture tends to accumulate on the inner surface rather than being effectively transferred to the outer layer^38^. The dense interlacing of the plain weave combined with the bulky roving weft likely leads to surface sealing and restricted moisture transport. Therefore, although the plain weave demonstrates mechanical robustness, it is not recommended for absorbent textile applications using roving weft yarns. Its potential use is limited to applications where mechanical stability is prioritized over moisture management such as reinforcement fabrics or barrier layers.

As the weft density increases from 5 to 9, the magnitude of the negative one-way transport index decreases, indicating a slight reduction in reverse moisture flow. However, the values remain negative, reflecting poor moisture transport performance in plain weave fabrics. This could be attributed to increased fiber packing restricting moisture absorption on the inner surface but not sufficiently enhancing outward movement.

In contrast, twill weave fabrics show significantly better performance, with positive OWTI values across all densities. At a weft density of 5, the index is 905.2%, remaining nearly constant at 904.5% for a density of 7, and slightly dropping to 806.2% at density 9. These high values confirm that twill structures facilitate efficient one-way moisture transport, which may be attributed to the more open and diagonal interlacing pattern characteristic of twill weaves. This structure allows capillary channels to form more effectively, promoting directional liquid movement.

The findings from the Overall Moisture Management Capacity (OMMC) further reinforce the observed trends in one-way transport behaviour between plain and twill weave fabrics. A higher OMMC value indicates superior moisture management performance compared to other woven fabrics.

For plain weaves, OMMC values decrease noticeably with increasing weft density, from 0.5 at 5 picks/cm to 0.15 at 9 picks/cm. This trend confirms that denser plain fabrics hinder moisture handling, likely due to the tighter and more compact yarn interlacements, which restrict liquid absorption and capillary action. In contrast, twill weaves exhibit much higher OMMC values, with 0.98 at 5 picks/cm and a gradual decline to 0.73 at 9 picks/cm. Even at the highest density, twill fabrics maintain substantially better moisture management compared to plain weaves. This supports the earlier observation that the diagonal and more open structure of twill facilitate better moisture spread and directional transfer, even under higher density conditions.

When comparing both fabric types, the results clearly demonstrate that twill weaves outperform plain weaves across all densities in terms of OMMC, aligning consistently with the One-Way Transport Index outcomes. In addition, the results from both the OWTI and the OMMC confirm that increasing weft density leads to a decline in moisture management performance.

**Table 5:** Results of OWTI and OMMC of fabric samples.

Sample code	One way transport index %	OMMC
P5	− 550.4 ± 11.39	0.5 ± 0.017
P7	− 480.9 ± 20.78	0.25 ± 0.02
P9	− 317.2 ± 9.45	0.15 ± 0.002
T5	905.2 ± 12.7	0.98 ± 0.011
T7	904.5 ± 19.9	0.78 ± 0.023
T9	806.2 ± 14.05	0.73 ± 0.005

The outcome underscores the innovative contribution of the current study, showing that T5, a twill weave structure, achieves a remarkable one-way transport index of 905%. This value represents a significant improvement over the traditional woven fabric based on a spun yarn blended of bamboo-banana fibers, which reached only 425%^38^. Moreover, Matusiak M., Sukhbat O^39^. presented a slightly higher index of 913.7% for a knitted fabric. It is well known that woven fabrics generally offer superior mechanical strength and structural integrity compared to knitted fabrics. Therefore, T5 effectively combines the durability advantages of woven fabrics with moisture transport performance approaching that of knitted fabrics.

## Conclusion

This study comprehensively evaluated the influence of weave structure and weft density on the physical, mechanical, tactile and absorption performance of a roving-weft cotton fabric. The findings revealed that fabric structure plays a critical role in determining properties such as air permeability, thermal conductivity, tensile strength, pilling resistance, roughness, and moisture management. Plain weave fabrics, especially at higher weft densities, demonstrated superior mechanical performance exhibiting higher tensile strength and pilling resistance, and lower warp-wise shrinkage. These outcomes are largely attributed to the higher number of interlacements in plain weaves, which improve yarn binding and structural integrity. The compact structure also increased inter-yarn friction, reducing yarn mobility and thus lowering elongation, particularly in the warp direction. On the other hand, twill weave fabrics showed notable advantages in tactile comfort, air permeability, and moisture management. The longer floats and fewer interlacements in twill structures facilitated greater airflow and more efficient vertical moisture transport through the fabric. These features contributed to a higher Overall Moisture Management Capacity (OMMC) and better performance on the One-Way Transport Index. In conclusion, although plain weaves demonstrate superior mechanical robustness, they fail to fulfil the primary functional requirement of moisture management, as evidenced by their negative OWTI values. Consequently, plain weaves are unsuitable specifically for applications requiring high one-way moisture transport but are suitable for applications prioritizing durability. In contrast, twill weaves-especially the T5 sample-exhibited the highest absorption performance and more effective moisture transport behaviour, making twill the only viable structural option for the functional absorbent application investigated in this study.

## Data Availability

The datasets used and/or analysed during the current study available from the corresponding author on reasonable request.
